# Early Echocardiographic Markers in Heart Failure with Preserved Ejection Fraction

**DOI:** 10.3390/jcdd12060229

**Published:** 2025-06-16

**Authors:** Annamaria Tavernese, Vincenzo Rizza, Valeria Cammalleri, Rocco Mollace, Cristina Carresi, Giorgio Antonelli, Nino Cocco, Luca D’Antonio, Martina Gelfusa, Francesco Piccirillo, Annunziata Nusca, Gian Paolo Ussia

**Affiliations:** 1 Department of Medicine and Surgery, Università Campus Bio-Medico di Roma, Via Alvaro del Portillo 21, 00128 Roma, Italy; an.tavernese@gmail.com (A.T.); giorgio.antonelli@unicampus.it (G.A.); a.nusca@policlinicocampus.it (A.N.); g.ussia@policlinicocampus.it (G.P.U.); 2 Hemodynamic Unit, Fondazione Policlinico Universitario Campus Bio-Medico, Via Alvaro del Portillo 200, 00128 Roma, Italy; n.cocco@policlinicocampus.it (N.C.); l.dantonio@policlinicocampus.it (L.D.); m.gelfusa@policlinicocampus.it (M.G.); f.piccirillo@policlinicocampus.it (F.P.); 3 Cardiovascular Imaging Unit, IRCCS San Raffaele Scientific Institute, Via Olgettina 60, 20132 Milan, Italy; rizza.vincenzo@hsr.it; 4 Department of Experimental Medicine, Università degli Studi di Roma Tor Vergata, Via Montpellier 1, 00133 Rome, Italy; rocco.mollace@uniroma2.it; 5 Cardiology Unit, Humanitas Gavazzeni, 24125 Bergamo, Italy; 6 Institute of Research for Food Safety & Health IRC-FSH, University Magna Graecia, 88100 Catanzaro, Italy; carresi@unicz.it

**Keywords:** heart failure with preserved ejection fraction (HFpEF), global longitudinal strain (GLS), left atrial strain (LAS), myocardial work (MW), right ventricular free wall longitudinal strain (RVFWLS), speckle-tracking echocardiography (STE), diastolic dysfunction, echocardiographic biomarkers, shear wave imaging (SWI), subclinical myocardial dysfunction

## Abstract

Heart failure with preserved ejection fraction (HFpEF) represents nearly half of all heart failure cases and remains diagnostically challenging due to its heterogeneous pathophysiology and often subtle myocardial dysfunction. Conventional echocardiographic parameters, such as left ventricular ejection fraction (LVEF) and the left atrial volume index (LAVI), frequently fail to detect early functional changes. Advanced echocardiographic techniques have emerged as valuable tools for early diagnosis and risk stratification. Global Longitudinal Strain (GLS) allows for the identification of subclinical systolic dysfunction, even with preserved LVEF. Left Atrial Strain (LAS), particularly reservoir and pump strain, provides sensitive markers of diastolic function and elevated filling pressures, offering additional diagnostic and prognostic insights. Myocardial Work (MW), through non-invasive pressure–strain loops, enables load-independent assessment of contractility, while Right Ventricular Free Wall Longitudinal Strain (RVFWLS) captures early right heart involvement, often present in advanced HFpEF. The integration of these advanced parameters can enhance diagnostic precision and guide personalized treatment strategies. This review highlights the current evidence and clinical applications of strain-based imaging in HFpEF, underscoring the importance of a multiparametric, pathophysiology-oriented approach in heart failure evaluation.

## 1. Introduction

Heart failure with preserved ejection fraction (HFpEF) accounts for nearly half of all heart failure (HF) cases, representing a growing global health concern [1]. Its prevalence continues to rise, driven by an aging population and an increasing burden of comorbidities such as hypertension, obesity, and metabolic syndrome [2]. Despite its high morbidity and an estimated 5-year mortality rate of up to 75%, diagnosing HFpEF remains challenging due to its complex pathophysiology and the limitations of conventional imaging techniques [3].

HFpEF is characterized by impaired left ventricular (LV) relaxation, increased LV stiffness, and elevated LV and left atrial (LA) filling pressures, leading to progressive pulmonary hypertension and right ventricular (RV) dysfunction [4]. Echocardiography remains the cornerstone for HF classification, distinguishing between HF with reduced (HFrEF) and preserved (HFpEF) ejection fraction. However, while echocardiographic assessment is the first pivotal step in characterizing diastolic dysfunction and estimating LV filling pressures, as recommended by the ASE/EACVI guidelines, it does not fully capture the multifaceted nature of HFpEF [5].

The growing prevalence of HFpEF-related risk factors, combined with limitations in conventional diagnostic tools, has fueled interest in novel echocardiographic parameters capable of detecting subclinical myocardial dysfunction and refining early diagnosis and risk stratification [2,6]. Among these, Global Longitudinal Strain (GLS) has emerged as a sensitive marker of early systolic impairment, even when left ventricular ejection fraction (LVEF) appears preserved [7]. Similarly, Left Atrial Strain (LAS) provides insights into atrial function and filling pressures, serving as an early indicator of diastolic dysfunction [8]. Myocardial Work (MW), which integrates pressure–strain analysis, allows for a load-independent assessment of myocardial performance [9]. Additionally, Right Ventricular Free Wall Longitudinal Strain (RVFWLS) plays a key role in evaluating RV function and pulmonary hypertension, which frequently coexist with HFpEF [10]. Lastly, Shear Wave Imaging (SWI) has emerged as a novel, non-invasive tool to quantify myocardial stiffness, providing insights into fibrosis and extracellular matrix remodeling [11]. While it has not yet gained traction in clinical practice, it holds promise as a valuable imaging tool for assessing fibrosis and extracellular matrix remodeling in this context.

By integrating these advanced echocardiographic parameters, clinicians can achieve earlier and more accurate diagnoses, improve risk stratification, and tailor personalized therapeutic strategies. This review explores the latest evidence supporting the clinical utility of GLS, LAS, MW, and RVFWLS in refining the diagnosis and management of HFpEF.

## 2. Limitations in the Diagnosis of HFpEF

According to the 2021 ESC guidelines on heart failure, HFpEF is diagnosed based on objective evidence of cardiac structural and/or functional abnormalities indicative of LV diastolic dysfunction and elevated LV filling pressures, including increased natriuretic peptide levels [12]. To improve diagnostic accuracy, various algorithms have been developed, incorporating clinical parameters, ECG findings, echocardiographic and laboratory markers, invasive hemodynamic measurements, and advanced imaging techniques to determine the underlying etiology [3,6]. However, conventional echocardiographic parameters and existing algorithms exhibit limitations so that diagnosing HFpEF remains a major clinical challenge, with approximately 15% of patients falling into a diagnostic “grey zone” due to suboptimal performance of traditional parameters and algorithms [13]. One of the primary limitations in HFpEF diagnosis is the widespread reliance on LVEF. While LVEF is universally accepted and easy to calculate, it has several limitations that reduce its diagnostic and prognostic utility, particularly in HFpEF. LVEF does not correlate with symptoms and fails to predict clinical outcomes, as it primarily reflects geometric changes in the LV rather than intrinsic contractile function or LV filling pressures [14,15]. A key limitation of LVEF is its inability to detect early systolic dysfunction, as it measures global ventricular function but overlooks subclinical myocardial abnormalities, which can instead be detected using GLS. Additionally, LVEF is highly dependent on loading conditions, with acute afterload changes (e.g., hypertension) reducing LVEF even in the absence of contractile dysfunction, while preload reductions (e.g., hypovolemia) can falsely improve LVEF values [16]. Moreover, LVEF predominantly assesses radial myocardial contraction, disregarding longitudinal and circumferential contractile components, which are more sensitive markers of myocardial impairment [17]. The diagnostic complexity of HFpEF is further exacerbated by its non-specific symptoms, which often overlap with those of other conditions, delaying recognition and appropriate management. A major challenge is the high prevalence of comorbidities that frequently accompany HFpEF, including obesity, chronic respiratory diseases, diabetes, and chronic kidney disease, all of which can alter cardiac function and mimic HF symptoms [18]. These non-cardiac comorbidities significantly influence prognosis and mortality risk, making it difficult to isolate the specific contribution of myocardial dysfunction [19]. Additionally, natriuretic peptides may not always be elevated, particularly in obese individuals or early disease stages, limiting their reliability as diagnostic markers [20]. Furthermore, atrial fibrillation (AF) is present in more than 50% of HFpEF patients, significantly affecting diastolic function assessment. AF alters left ventricular filling patterns due to the absence of atrial systole, leading to increased left atrial pressure and further complicating the application of standard echocardiographic criteria for HFpEF diagnosis [21]. The presence of AF necessitates different echocardiographic approaches for evaluating diastolic dysfunction, further adding complexity to the diagnostic process. A comprehensive diagnostic approach, integrating advanced echocardiographic parameters, hemodynamic assessments, and comorbidity profiling, is essential for improving HFpEF diagnosis and management.

## 3. Global Longitudinal Strain (GLS)

**Figure 1:**
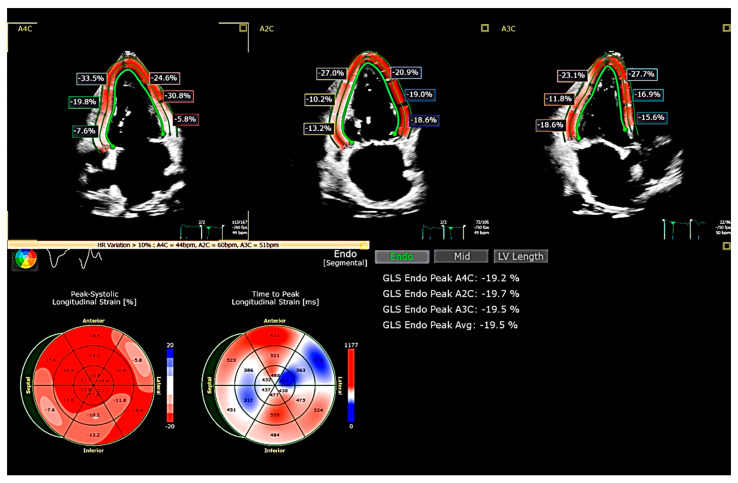
LV GLS (assessed using speckle-tracking echocardiography) of a patient affected by dilated cardiomyopathy with borderline low ejection fraction (LVEF 51%) but significantly impaired longitudinal contraction of basal septum, basal lateral, and mid-basal inferior walls. GLS = Global Longitudinal Strain; LV = Left Ventricle; LVEF = Left Ventricular Ejection Fraction.

GLS has become a key parameter in the evaluation of HFpEF, offering superior sensitivity over LVEF in detecting subclinical myocardial dysfunction (Figure 1, Supplementary Video S1). Initially developed as a Doppler-based technique, strain imaging has evolved with speckle-tracking echocardiography (STE), which is now the clinical standard due to its angle independence and better reproducibility [22,23]. GLS is calculated as the average peak systolic longitudinal strain across all LV segments obtained from apical four-, three-, and two-chamber views [24].

There is currently no universally accepted lower limit for GLS, as normal values vary depending on the vendor. Studies have shown that GLS is influenced by factors such as age, sex, body weight, and blood pressure. However, a meta-analysis of individual patient data suggests that an absolute GLS below 16% is a strong indicator of significant myocardial dysfunction, regardless of vendor differences or clinical covariates. This aligns with a recent study that identified 15.7% as the lower limit of normal in a large cohort of healthy individuals using standardized chamber-specific recordings, minimizing variability [25].

The mechanistic differences between GLS and LVEF explain why LVEF often fails to detect early myocardial dysfunction in HFpEF. LVEF is largely influenced by radial and circumferential myocardial contraction, whereas GLS primarily reflects longitudinal fiber shortening, which is predominantly driven by the subendocardial fibers. These fibers are particularly susceptible to ischemic and hemodynamic stress, making GLS a more sensitive marker of myocardial dysfunction than LVEF [17,26]. Moreover, LV hypertrophy, a frequent characteristic of HFpEF, results in reduced cavity size, meaning that even a small stroke volume can yield a normal or supranormal LVEF, further masking underlying contractile dysfunction [27].

The clinical implications of subclinical systolic dysfunction in HFpEF have been further validated by findings from the PARAGON-HF study. This study demonstrated that GLS impairment is strongly associated with adverse clinical outcomes, including worsening HF symptoms and increased hospitalization risk, even in patients with preserved LVEF [28]. Similarly, impaired longitudinal systolic function has been documented in other HFpEF cohorts and was associated with adverse clinical outcome, further highlighting its prognostic relevance [7,29,30]. These findings underscore the need for GLS to be routinely incorporated into HFpEF assessment, as it provides independent prognostic value beyond LVEF.

Despite its advantages, GLS measurement is not without limitations. Two-dimensional STE (2D-STE) remains the standard technique; however, it is affected by geometric assumptions, foreshortened apical views, and tracking challenges due to out-of-plane speckle motion. The development of three-dimensional STE (3D-STE) overcomes many of these limitations by allowing for a more comprehensive and accurate assessment of myocardial function. Notably, 3D-STE has demonstrated incremental prognostic value over 2D-STE in HFpEF populations, though further validation in large cohorts is still needed [31].

Overall, GLS represents a powerful diagnostic and prognostic tool in HFpEF, offering greater sensitivity than LVEF for detecting early myocardial dysfunction. As imaging technologies evolve, 3D-STE may further refine GLS assessment, providing a more precise evaluation of myocardial function and enhancing risk stratification in HFpEF patients. Moreover, some “GLS patterns” have been attributed a significant diagnostic value in specific cardiac diseases (Table 1).

## 4. Myocardial Work (MW)

**Figure 2:**
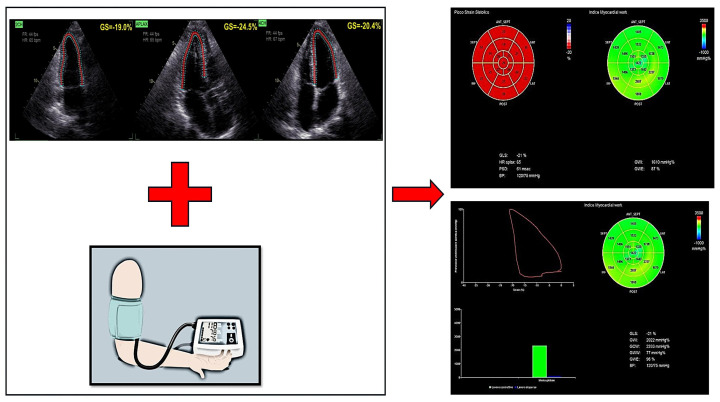
LV Myocardial Work (MW) indices calculated combining LV GLS and peak arterial pressure evaluated with a cuff manometer. FR = Frame Rate; BP = Blood Pressure; GCW = Global Constructive Work; GLS = Global Longitudinal Strain; GS = Global Strain; GWE = Global Work Efficiency; GWI = Global Work Index; GWW = Global Wasted Work; HR = Heart Rate; LV = Left Ventricle; PSD = Peak Strain Dispersion.

Myocardial Work (MW) has emerged as an advanced echocardiographic tool that addresses the limitations of Global Longitudinal Strain (GLS), particularly its dependency on loading conditions. Historically, pressure–volume loop analysis derived from cardiac catheterization played a fundamental role in quantifying myocardial effort and oxygen consumption, providing crucial insights into preload, afterload, and contractility. However, despite its value, this invasive approach is not feasible for routine clinical practice [37]. To overcome these challenges, a non-invasive echocardiographic method for assessing MW was developed and validated in 2018, allowing for the integration of LV strain analysis with estimated ventricular pressure. This technique calculates MW indices by incorporating cuff-measured blood pressure, offering a more load-independent assessment of LV function compared with GLS (Figure 2). A vendor-specific methodology (GE Healthcare, Pewaukee, WI, USA) was introduced to estimate peak LV pressure non-invasively, enabling a more precise evaluation of myocardial efficiency.

MW is quantified using four primary indices:

- Global Work Index (GWI)—reflects the total myocardial effort performed by the LV.
- Global Constructive Work (GCW)—represents the portion of myocardial effort contributing to effective contraction.
- Global Wasted Work (GWW)—indicates inefficient myocardial effort due to dyssynchronous or ineffective contraction.
- Myocardial Work Efficiency (MWE)—evaluates the ratio of constructive to total myocardial effort, serving as an indicator of LV contractile performance.

Reference values for Myocardial Work (MW) indices have been established and are available in the work by Manganaro et al. [38].

These indices have been validated in large echocardiographic cohorts as reliable markers of ventricular contractile function [9].

Several studies have highlighted the clinical relevance of MW in HFpEF. D’Andrea et al. found that patients with HFpEF exhibit impaired MW efficiency, with higher levels of GWW and lower GCW and GWE, suggesting inefficient myocardial contraction despite preserved LVEF [39]. Similarly, a study by Guo et al. demonstrated that MW parameters are altered even in early stages of HFpEF, correlating with diastolic dysfunction and LV stiffness [40].

The ability of MW indices to improve HFpEF risk stratification has also been supported by prospective studies. Paolisso et al. showed that non-invasive MW assessment is predictive of first hospitalization in de novo HFpEF, emphasizing its potential role in early risk detection [41]. Additionally, Lin et al. explored the relationship between LV geometry and MW, finding that concentric LV remodeling in HFpEF is associated with increased myocardial wasted work, a factor linked to worse functional capacity and outcomes [42].

From a diagnostic perspective, MW also improves the differentiation between HFpEF and other forms of heart failure. Lan et al. demonstrated that combining MW indices with speckle-tracking strain analysis provides a more comprehensive evaluation of LV function in HFpEF patients, compared with strain parameters alone [43].

Beyond its diagnostic value, MW may also offer prognostic insights into therapy response. Galli et al. investigated the role of MW in predicting response to cardiac resynchronization therapy (CRT), suggesting that constructive work could identify patients who would benefit most from treatment [44]. Though this study was conducted in HFrEF, the concept of MW-guided therapy selection could be extrapolated to HFpEF management, particularly in identifying patients who may respond better to device-based or pharmacologic interventions.

In conclusion, MW represents an advanced echocardiographic tool for HFpEF evaluation, providing load-independent insights into myocardial efficiency and contractility. Its ability to predict HFpEF progression, hospitalizations, and response to therapy suggests that incorporating MW into routine echocardiographic assessment could improve patient stratification and management strategies. However, its clinical adoption still requires awareness of certain methodological limitations, as discussed below.

Despite its growing clinical utility, Myocardial Work (MW) is not without limitations. It relies on the non-invasive estimation of left ventricular pressure using brachial cuff systolic blood pressure, which may not accurately reflect central or true LV pressure—particularly in patients with arterial stiffness, peripheral vascular disease, or pressure amplification. Moreover, MW indices depend on accurate identification of valvular timing events and high-quality apical image acquisition, both of which can impact reproducibility. These factors may limit feasibility in certain patient populations and underscore the importance of interpreting MW within a broader, multiparametric diagnostic framework [9,37,38].

## 5. Right Ventricular Free Wall Longitudinal Strain (RVFWLS)

**Figure 3:**
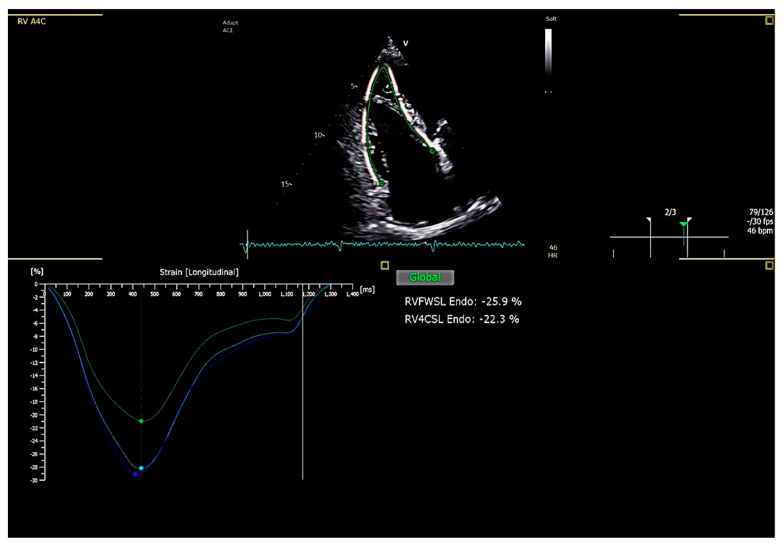
Right Ventricular Free Wall Longitudinal Strain (assessed using speckle-tracking echocardiography). RVFWLS = Right Ventricular Free Wall Longitudinal Strain; RV4CLS = Right Ventricular 4-Chamber Longitudinal Strain.

Advances in imaging technology and the growing body of clinical evidence have increasingly highlighted the importance of right ventricular (RV) strain assessment in cardiovascular medicine. In particular, RV longitudinal strain has emerged as a reliable and sensitive tool for evaluating RV systolic function across a variety of myocardial and valvular pathologies, offering incremental diagnostic and prognostic information beyond conventional echocardiographic parameters [45]. For accurate and reproducible measurements, RV-focused apical four-chamber views are recommended to optimize visualization and tracking (Figure 3; Supplementary Video S2) [46].

Among the different strain parameters, RV free wall longitudinal strain (RVFWLS)—which specifically assesses deformation of the three free wall segments while excluding the interventricular septum—is currently the most widely used in clinical practice. This parameter has shown greater sensitivity and prognostic value compared with global RV strain. Data from large cohorts of healthy individuals have suggested that absolute RVFWLS values below 20% are indicative of subclinical dysfunction [47,48], with more recent evidence identifying 17% as the lower limit of normal [49].

In HFpEF, RV dysfunction results from a combination of increased left-sided filling pressures, pulmonary hypertension, and potentially intrinsic myocardial abnormalities. T [50,51]. Recent findings from the PARAGON-HF study [52] have suggested that impaired RVFWLS and disrupted right ventricular–pulmonary arterial coupling are independently associated with more advanced heart failure symptoms and worse cardiovascular outcomes, irrespective of left ventricular function.

Nevertheless, RV strain assessment presents specific technical challenges. The complex geometry of the RV, its thin myocardial wall, and strong dependence on loading conditions make accurate strain quantification difficult. Reliable tracking requires optimal acoustic windows and apical alignment, which may be suboptimal in patients with obesity, arrhythmias, or chronic lung disease. Furthermore, interobserver variability remains significant despite growing standardization efforts [45,46,48]. These limitations must be acknowledged when interpreting RVFWLS in clinical practice and underscore the need for multiparametric integration.

## 6. Left Atrial Strain (LAS)

**Figure 4:**
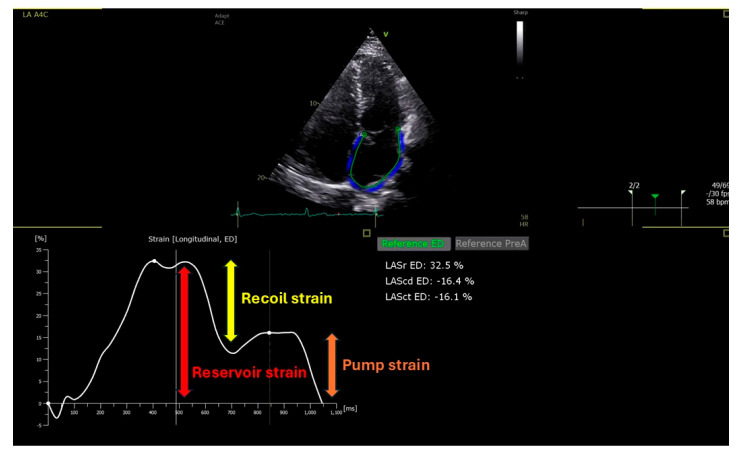
LA strain components. ED = End Diastole; LA = Left Atrium; LAScd = Left Atrial Recoil Strain; LASct = Left Atrial Pump Strain; LASr = Left Atrial Reservoir Strain.

Evaluation of diastolic dysfunction is a key component of echocardiographic heart failure assessment. In the current clinical landscape—where HFpEF is increasingly common and diastolic function remains challenging to evaluate—LAS has emerged as a promising diagnostic parameter. Although recent diastolic function imaging guidelines have been revised to be more straightforward, the current 2016 recommendations by the American Society of Echocardiography (ASE) and the European Association of Cardiovascular Imaging (EACVI) can still be difficult to apply in routine clinical practice [5]. The echocardiographic assessment of diastolic function typically involves measuring early diastolic mitral annular velocity (e’), the E/e’ ratio, the left atrial volume index (LAVI), and tricuspid regurgitation velocity (TRV). Previous studies have shown that LAS is reduced in individuals with HFpEF [53], and it has been associated with invasive measures of diastolic function and left ventricular filling pressures [54]. Consistent with these findings, additional studies have demonstrated that left atrial reservoir strain is capable of identifying left ventricular diastolic dysfunction and elevated filling pressures even in cases where the LAVI remains within normal limits [55]. Importantly, LA strain comprises different components reflecting distinct phases of atrial function: reservoir strain—reflecting atrial filling during LV systole; recoil strain—indicating the passive LA contraction in early diastole (when blood flow is directed from the pulmonary veins towards the LV); and pump strain—representing the active atrial contraction in late diastole (Figure 4; Supplementary Video S3). Although these components are influenced by LV filling pressure, they also respond differently to changes in preload, afterload, and atrial contractility. For instance, while reservoir strain is more strongly affected by LV systolic function and atrial compliance, pump strain is sensitive to atrial contractility and may be unreliable in the presence of arrhythmias or atrial myopathy [56]. In terms of reference values, median normal values have been reported around 42% for LA reservoir strain and 14% for LA pump strain, with lower limits of normal considered to be 17% and 6%, respectively. However, in clinical decision making, a reservoir strain value < 20–23% is commonly considered abnormal, and values < 16–18% may be indicative of elevated LV filling pressures, especially when pulmonary capillary wedge pressure exceeds 12–15 mmHg. For LA pump strain, values < 8% in middle-aged and older adults are typically considered suggestive of elevated filling pressures. Notably, high-normal values of LA pump strain (>14%) combined with preserved LV Global Longitudinal Strain (GLS) (>18%) strongly predict normal LV filling pressure [49,57]. Regarding recoil strain, as it represents the difference between LA reservoir strain and pump strain, the need to assess this measurement as a distinct parameter is questionable [25]. While the LAVI remains a widely used structural marker of chronically elevated filling pressures, it has limited sensitivity for identifying early increases in left ventricular filling pressure. Recent evidence indicates that combining the LAVI with a dynamic functional parameter such as LA reservoir strain significantly improves the detection of diastolic dysfunction and elevated filling pressures in patients with preserved ejection fraction, compared with relying on the LAVI alone [58]. Not only has an association between atrial strain and diastolic dysfunction been demonstrated but also a correlation with its severity. In particular, Singh et al. observed that LA strain progressively declined in parallel with worsening diastolic function, offering a reliable method for grading the severity of dysfunction [59]. Moreover, studies have indicated that lower Left Atrial Strain (LAS) is linked to poorer clinical outcomes, highlighting its significance as a prognostic indicator [60,61]. Nonetheless, the use of LAS in clinical practice requires awareness of its technical and physiological limitations. LAS measurement is influenced by several hemodynamic and structural factors. It is sensitive to preload conditions, meaning that acute volume shifts can affect strain values independently of intrinsic atrial function [54,56]. Additionally, the presence of atrial fibrillation—common in HFpEF—precludes reliable assessment of pump strain and alters reservoir strain dynamics [47]. Structural remodeling of the left atrium, particularly fibrosis, can further compromise LAS accuracy [61]. These confounders highlight the importance of integrating LAS with other echocardiographic parameters and clinical data, rather than using it as a standalone diagnostic tool.

## 7. Future Perspectives

A promising technique for the future in the field of cardiac imaging is Shear Wave Imaging (SWI). SWI is an ultrasound-based elastography method that measures the propagation speed of mechanically induced shear waves within the myocardium, which directly correlates with myocardial stiffness. This parameter is particularly relevant in conditions characterized by increased fibrosis and extracellular matrix remodeling, such as HFpEF, providing real-time mechanical characterization of myocardial tissue, potentially allowing early detection of diastolic dysfunction [11].

Several preclinical studies have demonstrated the feasibility and accuracy of SWI in quantifying myocardial stiffness, validating the technique against gold-standard methods such as invasive pressure–volume loops and magnetic resonance elastography [62,63]. Additionally, early clinical studies have confirmed the ability of SWI to differentiate between healthy myocardium and pathological conditions associated with increased stiffness, supporting its potential role as a non-invasive biomarker for myocardial fibrosis [11].

In the specific context of HFpEF, the PACIFIC-PRESERVED study has been designed to further explore the clinical utility of SWI [64]. This ongoing multicenter trial aims to characterize myocardial mechanical properties in patients with HFpEF and to integrate SWI into a broader multiparametric imaging approach for patient phenotyping and risk stratification. The results of this study are expected to define the clinical role of SWI and its potential to improve diagnostic accuracy in HFpEF populations. However, it is important to note that SWI is not without limitations. Two main modalities are currently used in cardiac SWI: acoustic radiation force impulse (ARFI) and transient elastography (TE). ARFI uses focused ultrasound beams to generate localized shear waves within the myocardium, which are tracked to calculate tissue stiffness. TE, on the other hand, employs low-frequency mechanical vibrations—often externally applied—to induce shear waves, allowing for the assessment of regional myocardial stiffness through ultrafast imaging. Despite their promising applications, both techniques remain limited by several technical and physiological factors. SWI is highly dependent on image quality and acoustic window, which can be suboptimal in certain patient populations. Moreover, shear wave velocity is sensitive to loading conditions, making it difficult to distinguish intrinsic myocardial stiffness from dynamic hemodynamic changes. Finally, the lack of standardized acquisition protocols and validated reference values currently limits the widespread clinical adoption of these methods [11,65,66]. Further technological refinement and large-scale validation will be essential to unlock the full potential of SWI in HFpEF evaluation.

## 8. Integration with Other Diagnostic Modalities

While this review focuses on echocardiographic imaging, it is important to acknowledge the complementary role of other diagnostic tools in the evaluation of HFpEF, particularly in its early or subclinical stages. Cardiac magnetic resonance (CMR) offers unparalleled tissue characterization capabilities and is increasingly employed to detect subclinical myocardial remodeling, such as diffuse interstitial fibrosis or extracellular matrix expansion, even before functional impairment becomes evident. Through techniques like T1 mapping and extracellular volume (ECV) quantification, CMR can reveal early pathological changes that may precede diastolic dysfunction or elevated filling pressures, thus playing a crucial role in identifying patients at risk of HFpEF progression [67,68].

In parallel, natriuretic peptides such as BNP and NT-proBNP remain essential biomarkers for diagnosis and prognostication. Elevated levels correlate with increased cardiac filling pressures and adverse outcomes, although their diagnostic sensitivity may be limited in obese individuals or in the early stages of disease, where values can remain within normal range despite incipient dysfunction [69,70].

A multiparametric approach that integrates echocardiographic strain imaging with biochemical and CMR-based indices of myocardial tissue health may significantly enhance early detection, diagnostic precision, and individualized care in patients with or at risk for HFpEF.

## 9. Conclusions

HFpEF is a multifaceted clinical entity, driven by complex and heterogeneous pathophysiological pathways that often elude conventional diagnostic approaches. Traditional echocardiographic parameters, such as left ventricular ejection fraction and standard diastolic indices, provide an incomplete picture, failing to capture the subtle myocardial and atrial alterations that precede overt dysfunction. In this evolving landscape, the integration of advanced echocardiographic modalities has the potential to transform the diagnostic paradigm. These tools allow for the detection of subclinical myocardial impairment, offer refined phenotypic characterization, and enhance the ability to predict clinical outcomes.

While the complementary value of GLS, MW, LAS, and RVFWLS is well recognized, practical limitations in real-world clinical settings—such as time constraints, operator dependency, and restricted software availability—necessitate a pragmatic prioritization. Among these tools, GLS stands out as the preferred first-line marker, owing to its widespread availability, relatively short acquisition and analysis time, and strong prognostic relevance. It enables early identification of subclinical systolic dysfunction, even in patients with preserved LVEF, offering a readily actionable parameter in the initial diagnostic workup of suspected HFpEF.

By contrast, MW provides more detailed pathophysiological insight and superior load independence, but requires additional steps—including brachial pressure input and vendor-specific software—which may hinder its routine application. In this context, a tiered and pragmatic approach appears most appropriate: starting with GLS, and integrating MW, LAS, and RVFWLS in more nuanced, diagnostically uncertain, or phenotypically complex cases. Such a strategy offers a balanced compromise between operational efficiency and diagnostic depth, while enabling more comprehensive patient characterization when clinically warranted.

However, the transition of these advanced parameters from research to routine clinical practice still faces several challenges. These include the standardization of acquisition and analysis protocols, the establishment of robust reference values across diverse populations and imaging platforms, and the need for large-scale prospective validation to confirm their prognostic and diagnostic utility. Overcoming these hurdles is essential for enabling broader clinical adoption. Ultimately, a multiparametric and pathophysiology-driven approach to cardiac imaging holds the potential not only to improve diagnostic precision in HFpEF but also to support more tailored and effective therapeutic decision making.

## Figures and Tables

**Table 1:** Examples of “GLS patterns” related to specific cardiac diseases.

GLS Pattern	Explanation	Cardiac Disease	Sensitivity and Specificity
Apical sparing	Normal or near-normal GLS values of LV apical segments associated with reduced GLS of basal and mid-ventricular segments	Cardiac amyloidosis	Se 93%, Sp 82% [32]
Global strain reduction	Uniformly low LV GLS values across all the ventricular segments	DCM chemotherapy-induced cardiac toxicity	Se 86%, Sp 70% [33] Se 70%, Sp 70% [34]
Basal accentuation	Hypercontractility of basal LV segments with higher GLS values in comparison with mid-apical segments	Takotsubo cardiomyopathy	Unknown precise Se and Sp [35]
“Cherry-on-top”	Hypercontractility of apical LV segments with higher GLS values in comparison with basal and mid-ventricular segments	Apical HCM	Se 93%, Sp 82% [36]

DCM = Dilated Cardiomyopathy; GLS = Global Longitudinal Strain; HCM = Hypertrophic Cardiomyopathy; LV = Left Ventricle; Se = Sensitivity; Sp = Specificity.

## Data Availability

The original data presented in the study can be found in the bibliographic references.

## Supplementary Materials

The following supporting information can be downloaded at: https://www.mdpi.com/article/10.3390/jcdd12060229/s1, Supplementary Video S1. LV GLS (assessed using speckle-tracking echocardiography) of a patient affected by dilated cardiomyopathy with borderline low ejection fraction (LVEF 51%) but significantly impaired longitudinal contraction of basal septum, basal lateral, and mid-basal inferior walls. GLS = Global Longitudinal Strain; LV = Left Ventricle; LVEF = Left Ventricular Ejection Fraction. Supplementary Video S2. Right Ventricular Free Wall Longitudinal Strain (assessed using speckle-tracking echocardiography). RVFWLS = Right Ventricular Free Wall Longitudinal Strain; RV4CLS = Right Ventricular 4-Chamber Longitudinal Strain. Supplementary Video S3. LA strain components. ED = End Diastole; LA = Left Atrium; LAScd = Left Atrial Recoil Strain; LASct = Left Atrial Pump Strain; LASr = Left Atrial Reservoir Strain.
